# Full-Fat Rice Bran Ameliorates Insulin Resistance and Modulates Muscle-Related Parameters in High-Fat Diet-Fed Ovariectomized Mice with Potential Involvement of the Gut–Muscle Axis

**DOI:** 10.3390/nu18111774

**Published:** 2026-05-30

**Authors:** Pei Yu Loe, Yusuke Ohsaki, Suh-Ching Yang, Hitoshi Shirakawa, Wan-Chun Chiu

**Affiliations:** 1 School of Nutrition and Health Sciences, College of Nutrition, Taipei Medical University, Taipei City 11031, Taiwan; da07113001@tmu.edu.tw (P.Y.L.); sokei@tmu.edu.tw (S.-C.Y.); 2 International Education and Research Center for Food Agricultural Immunology, Graduate School of Agricultural Science, Tohoku University, 468-1 Aramaki Aza Aoba, Aoba-ku, Sendai 980-8572, Japan; yusuke.ohsaki.a4@tohoku.ac.jp (Y.O.); shirakah@tohoku.ac.jp (H.S.); 3 Laboratory of Nutrition, Graduate School of Agricultural Science, Tohoku University, 468-1 Aramaki Aza Aoba, Aoba-ku, Sendai 980-8572, Japan; 4 Research Center of Geriatric Nutrition, College of Nutrition, Taipei Medical University, Taipei City 11031, Taiwan; 5 Department of Nutrition, Wan Fang Hospital, Taipei Medical University, Taipei City 11696, Taiwan

**Keywords:** full-fat rice bran, insulin resistance, muscle atrophy, gut microbiota, short-chain fatty acids, ovariectomized mice, high-fat diet

## Abstract

**Objectives:** The study aimed to evaluate the effects of full-fat rice bran (FFRB; Tainung No. 81, Taiwan) at various doses on insulin resistance, muscle atrophy, and gut microbiota composition in middle-aged ovariectomized (OVX) mice fed a high-fat diet (HFD), using young sham-operated mice as a life-stage reference group. **Methods:** Thirty-six female ICR mice were assigned to six groups, including OVX mice fed HFD with or without 5%, 10%, or 20% FFRB. **Results:** Compared with HFD-fed OVX controls, 20% FFRB reduced body weight gain by 43%, decreased visceral fat mass, and improved insulin resistance (homeostasis model assessment of insulin resistance, HOMA-IR reduced by 65%, *P_trend_* = 0.001). FFRB attenuated the decline in relative grip strength (forelimb, *P_trend_* = 0.013; four-limb, *P_trend_* < 0.001), and upregulated muscle protein synthesis genes, including insulin receptor substrate 1 (IRS-1), mammalian target of rapamycin (mTOR), eukaryotic translation initiation factor 4E binding protein 1 (eIF-4EBP1), while downregulating forkhead box protein O1 (FOXO1), muscle RING-finger protein-1 (MuRF-1), and interleukin (IL)-6. FFRB was also associated with higher fecal acetate levels (*P_trend_* < 0.001), upregulated colonic tight junction genes (occludin and zonula occludens (ZO)-1), and greater relative abundance of *g_Muribaculum*. Correlation analyses revealed positive associations between short-chain fatty acids (SCFAs) and muscle strength, muscle anabolic markers, genus *Lachnospiraceae_UCG_001*, and *Muribaculum*. **Conclusions:** Dietary inclusion of FFRB was associated with favorable metabolic and muscle-related parameters in HFD-fed middle-aged OVX mice, with potential involvement of gut microbiota and SCFA alterations.

## 1. Introduction

Postmenopause is when menstruation has ceased for over a year. Women normally experience menopause between the ages of 45 and 55 due to estrogen deficiency [1]. The decline in estrogen levels promotes visceral fat accumulation and may lead to obesity, glucose intolerance, and dyslipidemia, collectively known as metabolic syndrome [2]. In women, muscle mass and function gradually decline after the age of 50, with an estimated loss of about 0.6% per year after menopause; this process exacerbates after the age of 60 [3]. Muscle strength also decreases by approximately 15% every decade [4]. Menopausal women are particularly prone to muscle loss compared to men of the same age due to reduced estrogen levels [5], particularly estradiol, which plays an important role in maintaining muscle mass and limiting inflammation-induced damage to skeletal muscle [6]. Estradiol receptors have been identified in skeletal muscle fibers [7], and thus, a decline in estrogen levels further accelerates the progression of muscle atrophy in postmenopausal women [8].

Transition to postmenopausal state marks a key point in life where the effects of natural aging and rapid hormonal decline occur together. This period is often associated with chronic low-grade inflammation and elevated circulating pro-inflammatory cytokines, such as interleukin-6 (IL-6) and tumor necrosis factor-α (TNF-α) [9]. High-fat diet (HFD)-induced obesity promotes insulin resistance and further worsens systemic inflammation, which has been shown to disrupt intestinal permeability [10], resulting in reduced gut microbial diversity and richness [11]. This gut dysbiosis exacerbates metabolic dysfunction and may impair protein turnover, thereby contributing to muscle atrophy during this transition through mechanisms involving inflammation, oxidative stress, and altered short-chain fatty acid (SCFA) production [12,13,14]. Acetate, propionate, and butyrate are the three major SCFAs produced by gut microbiota, predominantly in the cecum and proximal colon [15]. Besides maintaining colonic health and microbial balance, SCFAs also influence glucose and energy homeostasis. Recent evidence has highlighted their protective effects against muscle atrophy via mitochondrial activity, glucose homeostasis, and insulin sensitivity, and reduced inflammation and oxidative stress [16].

Muscle mass is tightly regulated by a balance between anabolic and catabolic signaling pathways, which control protein synthesis and degradation. Insulin and insulin-like growth factor 1 (IGF-1) support muscle growth through the IGF-1/Akt /protein kinase B and mechanistic target of rapamycin (mTOR) pathways, which regulate muscle mass and protein synthesis [17,18]. In contrast, muscle atrophy is driven by interleukins activating the nuclear factor kappa B (NF-κB) pathway and by the upregulation of key ubiquitin E3 ligases, such as MAFbx/atrogin-1 and muscle RING-finger protein-1 (MuRF-1) [17,18,19]. Other pathways, including p38 mitogen-activated protein kinases (p38MAPK), NF-κB, forkhead box protein O (FOXO), and Janus kinase-signal transducer and activator of transcription (JAK-STAT), are also implicated in promoting muscle degradation [17]. These findings suggest that promoting anabolic activity while suppressing inflammation may serve as an effective intervention to mitigate muscle atrophy.

Rice is an important staple food consumed worldwide, especially in Asia [20]. Rice bran, a brown outer layer of rice kernel, is a by-product of rice milling [20], with global production reaching nearly 23.80 million tons in 2014–2015 [21]. Although rice bran is often utilized as compost or animal feed [22] due to its rapid rancidification induced by the lipase enzymes [21,22], rice bran is nutritionally dense and rich in dietary fiber and phytosterols, tocotrienols and γ-oryzanol, which have been shown to lower blood pressure, improve blood lipid and insulin sensitivity, reduce inflammation and oxidative stress [22,23], regulate gut microbiota by fostering the growth of fiber-degrading, SCFAs-producing bacteria [24,25], and protect against muscle atrophy [25,26,27,28]. Our previous study demonstrated that a 12-week intervention with 10% semi-defatted mixture of Koshihikari and Hitomebore rice bran effectively maintained muscle mass in HFD-fed ovariectomized (OVX) mice by downregulating atrogin-1 and MuRF-1 expressions [26]. However, the effects of local Taiwan rice bran on skeletal muscle health during transition into postmenopausal conditions remained unexplored. Therefore, this study aimed to evaluate the effects of different doses of Tainung No. 81 (TNG81) full-fat rice bran (FFRB) on insulin resistance, muscle atrophy, and gut microbiota composition in HFD-fed middle-aged OVX mice, using young sham-operated mice as a reference control.

## 2. Materials and Methods

### 2.1. Rice Bran Preparation

TNG81 FFRB was provided by Professor Suh-Ching Yang’s lab. The FFRB was stabilized by heating at 120 °C for 10 min to inactivate the lipase and lipoxygenase enzymes, thereby preventing it from becoming rancid [29,30]. The heated FFRB was cooled to room temperature and then stored at −80 °C. The nutrient contents were analyzed by SGS Taiwan (New Taipei City, Taiwan).

### 2.2. Animals and Study Design

Six 10-week-old young sham female ICR mice and 30 adult female ICR ovariectomized (OVX) mice (24–28 weeks old) were purchased from BioLASCO Taiwan Co., Ltd. (Yilan County, Taiwan). The young sham mice were included as a life-stage reference group to characterize baseline physiological parameters during adulthood. All mice were housed in Taipei Medical University Animal Centre under controlled conditions (23 ± 2 °C, 50% ± 10% humidity) with a 12/12 h light/dark cycle, with ad libitum access to food (Cat. No. 5001, Laboratory Diet, St. Louis, MO, USA) and water for one week of acclimation. This study was conducted in compliance with the 3R principles and approved by the Institutional Animal Care and Use Committee (IACUC) of Taipei Medical University (approval no. LAC2024-0031), with careful consideration of animal welfare. Bilateral ovariectomy was performed by trained professionals at BioLASCO to ensure refined surgical procedures and minimize animal distress.

The mice were divided randomly into six groups: (1) young sham mice fed a control diet (YC), (2) OVX mice fed a control diet (OC), (3) OVX mice fed a high-fat diet (OH), (4) OVX mice fed a HFD with 5% rice bran (OHR5), (5) OVX mice fed a HFD with 10% rice bran (OHR10), and (6) OVX mice fed a HFD with 20% rice bran (OHR20). The diet compositions are shown in Table 1. The fat content was 9.5% in the control diet and 45% in the HFD. Body weight was measured weekly, and food intake was measured every two days. At week 11, fecal samples were collected continuously for one week and stored at −80 °C until analysis. At week 12, all mice were fasted for 14 h prior to sample collection. The animals were then anesthetized and euthanized via cardiac puncture. Blood samples were collected, and liver, visceral adipose tissues (perirenal and peri-uterine fat), cecum, colon, uterus, gastrocnemius (GAS) muscle, and cecal luminal contents were excised, weighed, and stored at −80 °C until further analysis.

### 2.3. Grip Strength Measurement

The grip strength of the forelimb and four limbs of the mice was assessed using a BIOSEB’s grip strength meter (Cat. No. BIO-GS4, Pinellas Park, FL, USA) at weeks 0 and 11. For each testing session, three consecutive measurements were obtained for each mouse, and the mean value of the three trials was calculated. To account for baseline variability, the change from baseline (Δ = week 11 − week 0) in relative grip strength (normalized to body weight) was calculated and compared among OH and OHR groups.

### 2.4. Intraperitoneal Glucose Tolerance Test

IPGTT was conducted once at week 11 for all mice. The mice were administered 1 g/kg body weight of 20% glucose (Sigma-Aldrich, Cas No. 50-99-7, St. Louis, MO, USA) via i.p. injection after a 14 h fast. Blood was collected from the tail vein at 0, 30, 60, 90, and 120 min and quantified using an Easi-Check blood glucose meter and strips (Cat. No. TD-4207, New Taipei City, Taiwan). The total area under the curve (AUC; mg/dL × min), including the baseline value at 0 min, was calculated using the trapezoid rule based on serial glucose measurements. AUC was then analyzed as a continuous summary endpoint according to the statistical approach described in the Section 2.9.

### 2.5. Plasma Biochemical Analysis

For plasma biochemical analysis, blood samples were collected immediately after euthanasia and centrifuged at 1500× *g* for 15 min to obtain plasma. Plasma samples were analyzed for fasting glucose (GLU), triglycerides (TG), and total cholesterol (TC) using an automated chemistry analyzer AU5800 (LEZEN Reference Lab, Taipei, Taiwan). The plasma insulin concentration was measured using an insulin ELISA kit (Mercodia Ultrasensitive Mouse, Cat. No.10-1249-01, Uppsala, Sweden). Insulin resistance was estimated using indexes of homeostasis model assessment of insulin resistance (HOMA-IR = [fasting glucose (mmol/L) × fasting insulin (μU/mL)]/22.5).

### 2.6. RT-qPCR Gene Expression Analysis

Total RNA from the gastrocnemius muscle and colon was extracted using TRIzol reagent (Thermo Fisher Scientific, Cat. No. 15596018, Waltham, MA, USA) and reverse-transcribed with RevertAid First Strand cDNA Synthesis Kit (Thermo Fisher Scientific, Cat. No. K1621, Waltham, MA, USA). RT-PCR of target genes was performed using Maxima SYBR Green/ROX qPCR Master Mix (2×) (Thermo Fisher Scientific, Cat. No. K0221, Waltham, MA, USA) with the QuantStudio 1 Real-Time PCR System (Applied Biosystems, Ref No. A40425, Foster City, CA, USA). The target mRNA expression levels were normalized to the reference glyceraldehyde-3-phosphate dehydrogenase (GAPDH) mRNA, and the fold change of expression (YC/OH/OHR vs. OC) was calculated using the 2^−ΔΔCT^ method. The target mRNA primer sequences are stated in Supplementary Table S1.

### 2.7. Short-Chain Fatty Acids Analysis

Referring to the previously described methods with minor modifications [26,31], fecal short-chain fatty acids (SCFAs) were extracted with ethyl acetate (Cas No. 141-78-6, Macron Fine Chemicals, Center Valley, PA, USA) after acidification with 0.5% phosphoric acid (Honeywell Specialty Chemicals Seelze GmbH, Cas No. 7664-38-2, Seelze, Germany) and analyzed by gas chromatography–mass spectrometry (GC–MS, 7820A/5977B, Agilent Technologies, Santa Clara, CA, USA) equipped with a Nukol™ capillary column 30 m × 0.25 mm, df 0.25 μm (24107, Supelco, Bellefonte, PA, USA). The conditions set in the GC program are shown in Supplementary Table S2. Data acquisition and quantification were performed using Agilent MassHunter Workstation software 13.0 (Agilent Technologies, Santa Clara, CA, USA). Peaks were identified and quantified by comparison with a standard (Volatile Free Acid Mix, Lot No. LRAC0113, Sigma-Aldrich, St. Louis, MO, USA).

### 2.8. Cecum Microbiota Analysis

For gut microbiota analysis, a randomly selected subset of five mice per group was used for cecal sample sequencing because of resource constraints related to microbiota analysis. Cecal luminal DNA was sequenced on the Illumina MiSeq 300PE platform (Illumina, San Diego, CA, USA) targeting the 16S ribosomal (r)DNA V3–V4 hypervariable regions (the data were performed by Genomics, New Taipei, Taiwan). Microbiome bioinformatics were performed using the QIIME 2 (v2019.10) pipeline and R software 4.2.0. Briefly, adapters and low-quality bases were removed using Trimmomatic (v0.39), and primers were trimmed with Cutadapt (v0.39). Sequence denoising, error correction, and chimera removal were performed using the DADA2 algorithm to infer exact amplicon sequence variants (ASVs). Taxonomic classification was conducted using a pre-fitted Naive Bayes Scikit-learn classifier trained on the SILVA 132 database with 99% similarity. To control for multiple comparisons, the Benjamini–Hochberg (BH) false discovery rate (FDR) method was applied. Differential abundance of bacterial taxa was analyzed using the Kruskal–Wallis test followed by the BH correction. To identify specific microbial biomarkers, linear discriminant analysis (LDA) effect size (LEfSe) was employed at multiple taxonomic levels (from phylum to genus). Taxa were considered significant if the LDA score was >3. Statistical significance was defined as adjusted *p* < 0.05.

### 2.9. Statistical Analysis

Data are expressed as mean ± standard deviation (SD). Normality was assessed using the Shapiro–Wilk test. Normally distributed variables were analyzed by one-way ANOVA across all six groups, followed by pre-specified pairwise comparisons based on the study design (YC vs. OC, OC vs. OH, OH vs. OHR5/OHR10/OHR20, and pairwise comparisons among the OHR groups) with Šidák’s adjustment. Variables that did not meet normality assumptions were analyzed using the Kruskal–Wallis test followed by Dunn’s multiple-comparison test. This framework was applied to all continuous endpoints, including IPGTT AUC. For prespecified endpoint families (plasma biochemical parameters, muscle-related gene expression, SCFAs, and gut microbiota relative abundance), raw *p*-values from all pre-specified pairwise comparisons were further adjusted within each family using the BH–FDR procedure to obtain q-values. Raw *p*-values are reported for transparency, and q < 0.05 was considered statistically significant. Dose–response relationships were evaluated across the OH, OHR5, OHR10, and OHR20 groups by treating FFRB dose as an ordered variable. For normally distributed variables, a linear trend was assessed using a parametric test for trend, whereas Spearman’s rank correlation was used for variables that did not meet normality assumptions. Raw *p*-values from trend analyses were further adjusted using the BH–FDR method to obtain q-values. Spearman correlation analyses were conducted within the OVX intervention groups (OH and OHR groups) to evaluate associations between SCFAs (acetate, propionate, and butyrate) and metabolic, muscle-related, and microbiota parameters under a shared pathological and dietary background, and to minimize confounding by major structural differences related to age, ovariectomy status, and dietary background. For each SCFA, raw *p*-values across tested outcomes were adjusted using BH-FDR, and q < 0.05 was considered statistically significant. All statistical analyses and graphical representations were performed using GraphPad Prism 10.6 (GraphPad Software, San Diego, CA, USA).

## 3. Results

### 3.1. Nutrient Composition of Full-Fat Rice Bran

As shown in Table 2, TNG81 FFRB was rich in protein (14%), fat (14%), and insoluble fiber (21%). Among the amino acids, aspartic acid and glutamic acid were the most abundant (Table 3).

### 3.2. Effect of Full-Fat Rice Bran on Obesity-Related Indices

**Figure 1:**
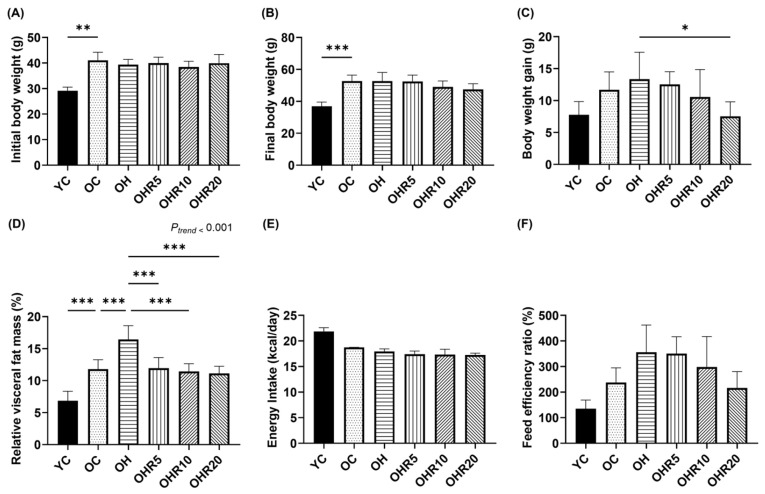
Effect of full-fat rice bran on obesity-related indices. (**A**) Initial body weight, (**B**) final body weight, (**C**) body weight gain, (**D**) relative visceral fat mass, (**E**) energy intake, and (**F**) food efficiency ratio (FER). Data are presented as mean ± SD (*n* = 6). The asterisk (*) indicates significant pairwise differences (*p* < 0.05 *, *p* < 0.01 **, *p* < 0.001 ***). Pre-specified group comparisons were conducted according to the Section 2.9. Variables shown in (**A**,**E**,**F**) were analyzed using the Kruskal–Wallis test followed by Dunn’s multiple-comparison test, whereas the other variables were analyzed using parametric tests. Dose-dependent effects across the intervention groups (OH and OHRs) are indicated by *P_trend_* values. Weight gain (g) = final body weight (g) − initial body weight (g). Relative visceral fat mass (%) = [visceral fat weight (g)/body weight (g)] × 100%. FER (%) = [average weight gain (g)/average food intake (g)] × 100%.

The initial and final body weights were significantly higher in the OC group than the YC group (Figure 1A,B), representing a descriptive contrast between the young sham-operated and adult OVX groups. As shown in Figure 1C, body weight gain was significantly lower in the OHR20 group than in the OH group. The relative visceral fat mass was higher in the OC group than in the YC group, higher in the OH group than in the OC group, while significantly lower in all OHR groups than in the OH group (Figure 1D). No significant differences were observed in energy intake or food efficiency ratio (FER) among the groups (Figure 1E,F).

### 3.3. Effect of Full-Fat Rice Bran on Lipid and Glucose Homeostasis

#### 3.3.1. Plasma Lipid Profile

**Figure 2:**
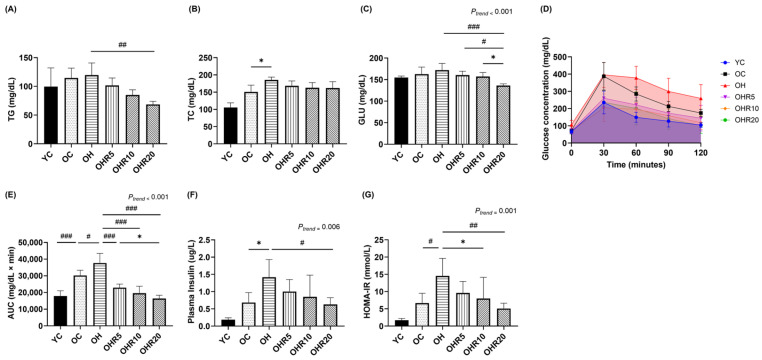
Effects of full-fat rice bran on lipid and glucose homeostasis. Lipid metabolism: plasma (**A**) TG, and (**B**) TC levels. Glucose metabolism: (**C**) plasma GLU level, (**D**) IPGTT, (**E**) AUC of glucose concentration, (**F**) plasma insulin concentration, and (**G**) HOMA-IR index. Data are presented as mean ± SD (*n* = 6). Pre-specified group comparisons were conducted according to the Section 2.9. Variables shown in (**A**,**B**) were analyzed using the Kruskal–Wallis test followed by Dunn’s multiple-comparison test, whereas the other variables were analyzed using parametric tests. The asterisk (*) indicates significant pairwise differences (*p* < 0.05 *). The hashtag (^#^) indicates statistical significance after Benjamini–Hochberg false discovery rate (BH-FDR) correction (q < 0.05 ^#^, q < 0.01 ^##^, q < 0.001 ^###^). Dose-dependent effects across the intervention groups (OH and OHRs) are indicated by *P_trend_* values after BH-FDR correction (q < 0.05).

Plasma TG levels were significantly lower in the OHR20 group compared with the OH group (Figure 2A). The reduction in TG remained statistically significant after BH–FDR correction for OH vs. OHR20 comparison. Plasma TC level was higher in the OH group than in the OC group (Figure 2B); this difference did not remain significant after BH-FDR correction.

#### 3.3.2. Glucose Profile

Fasting plasma GLU levels were reduced in the OHR20 group compared with the OH, OHR5, and OHR10 groups (Figure 2C). After BH-FDR correction, the OH vs. OHR20 and OHR5 vs. OHR20 comparisons remained significant, whereas the OHR10 vs. OHR20 comparison did not. A significant dose-dependent decrease in plasma GLU levels was observed across the intervention groups (*P_trend_* < 0.001). The intraperitoneal glucose tolerance test (IPGTT) showed that glucose concentrations in all groups peaked at 30 min and then decreased gradually. The OH group showed higher glucose levels and slower glucose clearance than the OC group, whereas all OHR groups displayed glucose spikes similar to the YC group (Figure 2D). The AUC of IPGTT was higher in the OC group than in the YC group, and higher in the OH group than in the OC group, while lower in all OHR groups than in the OH group, and lower in the OHR20 group than in the OHR5 group (Figure 2E). After BH–FDR correction, all comparisons remained significant except for OHR5 vs. OHR20. A significant dose-dependent decrease in AUC was observed across the intervention groups (*P_trend_* < 0.001). Plasma insulin level was higher in the OH group than in the OC and OHR20 groups (Figure 2F). After BH–FDR correction, the OH vs. OHR20 comparison remained significant, whereas the OC vs. OH comparison did not. A significant dose-dependent decrease was also observed (*P_trend_* = 0.006). HOMA-IR was higher in the OH group than in the OC group, and lower in the OHR10 and OHR20 groups than in the OH group (Figure 2G). After BH–FDR correction, the OC vs. OH and OH vs. OHR20 comparisons remained significant, whereas the OH vs. OHR10 comparison did not. A significant dose-dependent decrease in HOMA-IR was also observed (*P_trend_* = 0.001).

### 3.4. Effect of Full-Fat Rice Bran on Muscle

#### 3.4.1. Grip Strength

**Figure 3:**
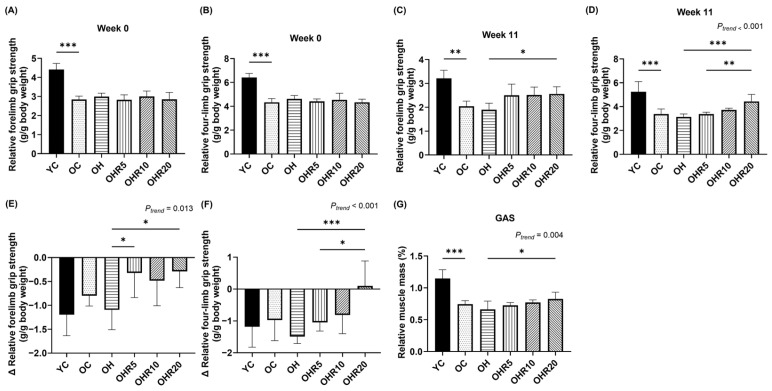
Effects of full-fat rice bran on muscle. Relative grip strength of (**A**) forelimb at week 0, (**B**) four-limb at week 0, (**C**) forelimb at week 11, and (**D**) four-limb at week 11. Change from baseline after 11 weeks of intervention in relative (**E**) forelimb and (**F**) four-limb grip strength. Negative values indicate loss of grip strength over time, whereas less negative values indicate reduced loss. (**G**) Relative GAS muscle mass. Data are presented as mean ± SD (*n* = 6). Pre-specified group comparisons were conducted according to the Section 2.9. Variable shown in (**C**) was analyzed using the Kruskal–Wallis test followed by Dunn’s multiple-comparison test, whereas the other variables were analyzed using parametric tests. The asterisk (*) indicates significant pairwise differences (*p* < 0.05 *, *p* < 0.01 **, *p* < 0.001 ***). Dose-dependent effects across the intervention groups (OH and OHRs) are indicated by *P_trend_* values. Relative GAS muscle mass (%) = [GAS muscle weight (g)/body weight (g)] × 100%.

At week 0, the OC group showed significantly lower relative forelimb and four-limb grip strength than the YC group, representing a descriptive contrast between the young sham-operated and adult OVX groups. No significant differences were observed among the OVX groups (Figure 3A,B). At week 11, relative forelimb and four-limb grip strength remained lower in the OC group than in the YC group. In contrast, the OHR20 group showed significantly higher forelimb grip strength than the OH group. Similarly, the OHR20 group exhibited higher four-limb grip strength than both the OH and OHR5 groups (Figure 3C,D). Relative forelimb and four-limb grip strength declined over time in most groups, whereas four-limb grip strength in the OHR20 group remained stable. When changes from baseline were analyzed, FFRB intervention significantly attenuated the loss of forelimb and four-limb grip strength in OVX mice in a dose-dependent manner (*P_trend_* = 0.013 and *P_trend_* < 0.001, respectively; Figure 3E,F).

#### 3.4.2. Muscle Mass

The OC group showed lower relative GAS muscle mass than the YC group, whereas the OHR20 group showed higher relative GAS muscle mass than the OH group (Figure 3G). A significant dose-dependent increase in relative GAS muscle mass was observed across the intervention groups (*P_trend_* = 0.004).

#### 3.4.3. Muscle Protein Synthesis Gene Expressions

**Figure 4:**
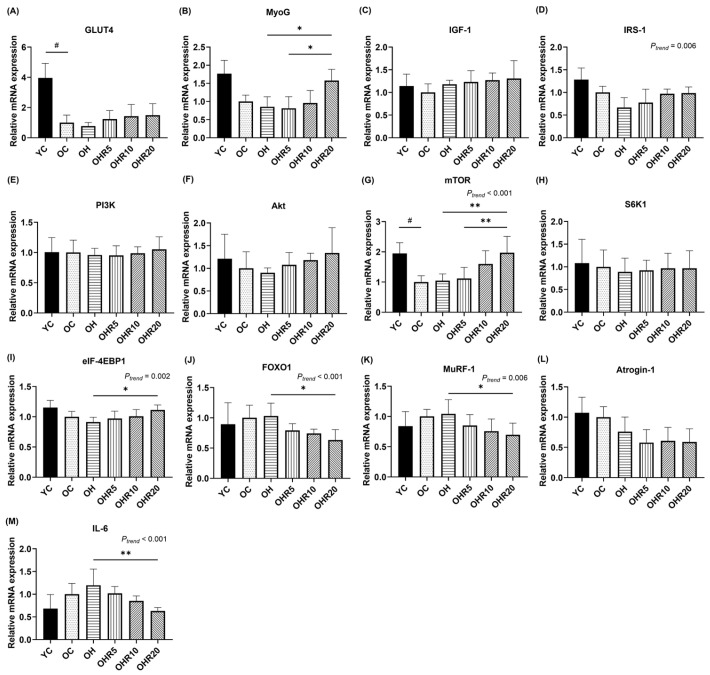
Effects of full-fat rice bran on muscle mRNA expressions related to protein synthesis (**A**–**I**), degradation (**J**–**L**), and inflammation (**M**) in HFD-induced middle-aged OVX mice. Data are expressed as mean ± SD (*n* = 6). Pre-specified group comparisons were conducted according to the Section 2.9. Variables shown in (**B**,**E**,**F**) were analyzed using the Kruskal–Wallis test followed by Dunn’s multiple-comparison test, whereas the other variables were analyzed using parametric tests. The asterisk (*) indicates significant pairwise differences (*p* < 0.05 *, *p* < 0.01 **). The hashtag (^#^) indicates statistical significance after Benjamini–Hochberg false discovery rate (BH-FDR) correction (q < 0.05 ^#^). Dose-dependent effects across the intervention groups are indicated by *P_trend_* values after BH-FDR correction (q < 0.05).

Figure 4A–I show the mRNA expression of genes involved in muscle protein synthesis signaling. The OC group showed lower mRNA expression of GLUT4 and mTOR than the YC group, and these differences remained significant after BH-FDR corrections. Compared with the OH group, the OHR20 group showed higher mRNA expression of MyoG, mTOR, and eIF-4EBP1. The OHR20 group also showed higher mRNA expression of MyoG and mTOR than the OHR5 group. However, these differences did not remain significant after BH-FDR correction. A significant dose-dependent increase was observed in the expression of IRS-1 (*P_trend_* = 0.006), mTOR (*P_trend_* < 0.001), and eIF-4EBP1 (*P_trend_* = 0.002) across the intervention groups, and these trends remained significant after BH-FDR correction (q < 0.05).

#### 3.4.4. Muscle Protein Degradation and Inflammatory Gene Expression Levels

Figure 4J–M show the mRNA expression of genes associated with muscle protein degradation and inflammation. The OHR20 group showed lower mRNA expression of FOXO1, MuRF-1, and IL-6 than the OH group, although these differences did not remain significant after BH-FDR correction. Dose–response analyses revealed significant decreasing trends in FOXO1 (*P_trend_* < 0.001), MuRF-1 (*P_trend_* = 0.006), and IL-6 (*P_trend_* < 0.001) across the intervention groups, which remained significant after BH-FDR correction (q < 0.05).

### 3.5. Effect of Full-Fat Rice Bran on Large Intestinal Barrier Function and Gut Microbiota

#### 3.5.1. Short-Chain Fatty Acids Production

**Figure 5:**
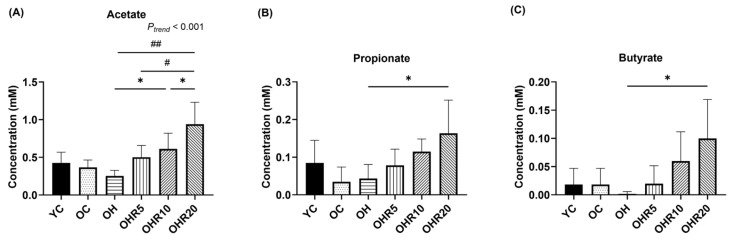
Effect of full-fat rice bran on the production of short-chain fatty acids. (**A**) acetate, (**B**) propionate, and (**C**) butyrate. Data are presented as mean ± SD (*n* = 6). Pre-specified group comparisons were conducted according to the Section 2.9. Variables shown in (**B**,**C**) were analyzed using the Kruskal–Wallis test followed by Dunn’s multiple-comparison test, whereas (**A**) were analyzed using parametric tests. The asterisk (*) indicates significant pairwise differences (*p* < 0.05 *). The hashtag (^#^) indicates statistical significance after Benjamini–Hochberg false discovery rate (BH-FDR) correction (q < 0.05 ^#^, q < 0.05 ^##^). Dose-dependent effects across the intervention groups are indicated by *P_trend_* values after BH-FDR correction (q < 0.05).

At the study endpoint, fecal acetate concentrations were higher in the OHR20 group than in the OH, OHR5, and OHR10 groups, and were also higher in the OHR10 group than in the OH group (Figure 5A). After BH-FDR correction, the OH vs. OHR20 and OHR5 vs. OHR20 comparisons remained significant, whereas the OH vs. OHR10 and OHR10 vs. OHR20 comparisons did not. Fecal propionate and butyrate concentrations were higher in the OHR20 group than in the OH group, although these differences did not remain significant after BH-FDR correction. A significant dose-dependent increase in fecal acetate concentrations was observed across the intervention groups (*P_trend_* < 0.001), and this trend remained significant after BH-FDR correction (q < 0.05).

#### 3.5.2. Intestinal Tight Junction

**Figure 6:**
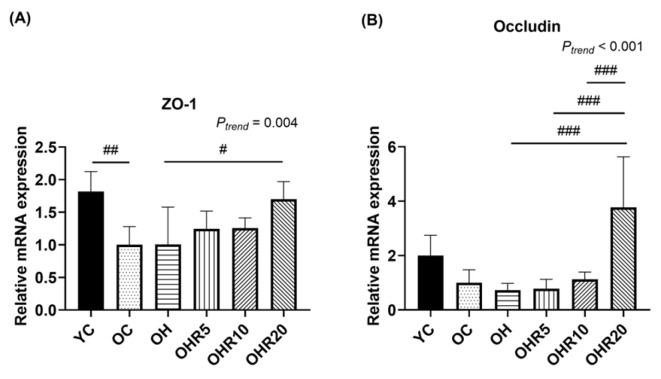
Effects of full-fat rice bran on intestinal permeability. The relative mRNA expression related to colonic tight junction: (**A**) ZO-1 and (**B**) occludin. Data are presented as mean ± SD (*n* = 6). Pre-specified group comparisons were conducted according to the Section 2.9. All variables were analyzed using parametric tests. The hashtag (^#^) indicates statistical significance after Benjamini–Hochberg false discovery rate (BH-FDR) correction (q < 0.05 ^#^, q < 0.05 ^##^, q < 0.05 ^###^). Dose-dependent effects across the intervention groups are indicated by *P_trend_* values after BH-FDR correction (q < 0.05).

Figure 6 shows the relative mRNA expression of colonic tight junction genes. ZO-1 expression was lower in the OC group than in the YC group, but higher in the OHR20 group than in the OH group, and these differences remained significant after BH-FDR correction. Occludin expression was higher in the OHR20 group than in the OH, OHR5, and OHR10 groups, and these differences remained significant after BH-FDR correction. A significant dose-dependent increase was observed in ZO-1 (*P_trend_* = 0.004) and occludin (*P_trend_* < 0.001) expression across the intervention groups, and these trends remained significant after BH-FDR correction (q < 0.05).

#### 3.5.3. Gut Microbiota Composition

**Figure 7:**
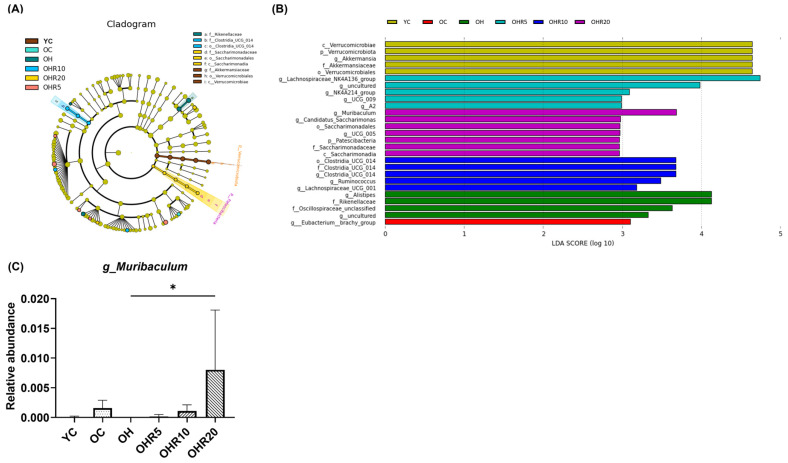
Effects of full-fat rice bran on gut microbiota composition. Gut microbiota analysis was performed on a randomly selected subset of five mice per group. (**A**) Linear discriminant analysis (LDA) effect size cladogram. (**B**) Histogram of the LDA scores. The significant difference among groups was detected by an LDA score ≥ 3. (**C**) The relative abundance of *g_Muribaculum*. Data are presented as mean ± SD (*n* = 5). Variable (**C**) was analyzed using the Kruskal–Wallis test followed by Dunn’s multiple-comparison test. The asterisk (*) indicates significant pairwise differences (*p* < 0.05 *). No pairwise comparisons remained significant after BH-FDR correction.

Figure 7 presents gut microbiota data based on a randomly selected subset of five mice per group. Exploratory taxonomic patterns across groups were assessed using linear discriminant analysis effect size (LEfSe). The cladogram and LDA score plot showed group-associated compositional features, with *Verrucomicrobiota* (notably *Akkermansiaceae*) more prominent in the YC group, [Eubacterium]_branchy_group in the OC group, with *Rikenellaceae*, *Alistipes*, and *Oscillospiraceae* in the OH group. In the OHR groups, taxa such as *Clostridia_UCG_014*, *Lachnospiraceae*, *Ruminococcus*, *Muribaculum*, and *Saccharimonadales* were descriptively enriched. The relative abundance of *g_Muribaculum* was higher in the OHR20 than in the OH group. However, because no microbiota features remained statistically significant after BH-FDR correction, these findings should be interpreted as descriptive and exploratory rather than as definitive evidence of FFRB-induced microbial alterations.

### 3.6. Correlation Between Short-Chain Fatty Acids, Blood Parameters, Muscle-Related Gene Markers, and Gut Microbiota

**Figure 8:**
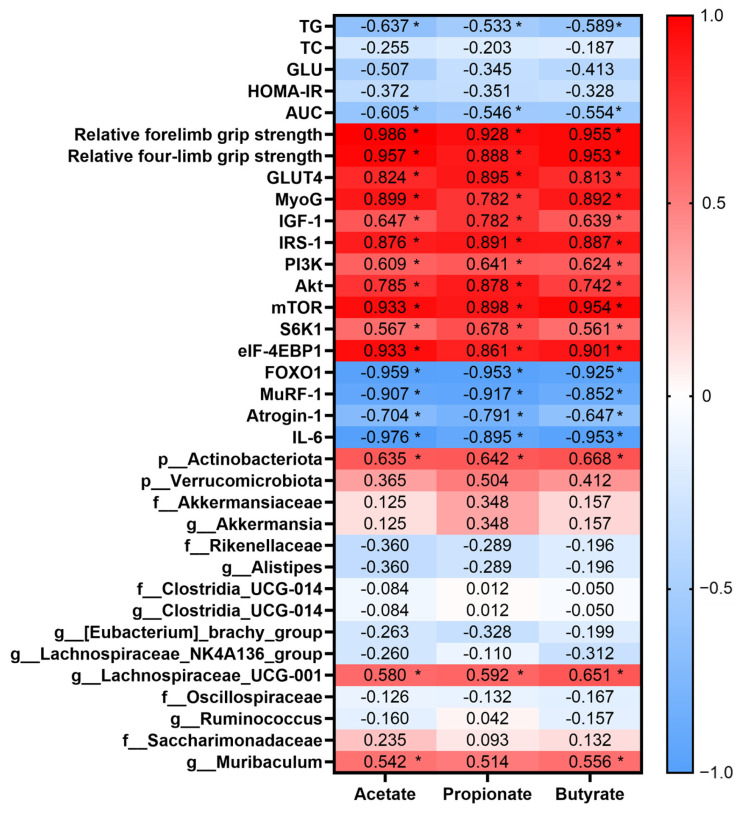
The correlation heatmap showing associations between SCFAs and physiological parameters. Spearman correlation coefficients (*r*) were calculated between fecal SCFAs (acetate, propionate, and butyrate) and plasma parameters, relative grip strength, muscle-related gene expression, and gut microbiota relative abundance within the intervention groups (OH and OHR groups). *p*-values were adjusted for multiple testing using the Benjamini–Hochberg false discovery rate (BH-FDR) method within each SCFA family of tests. The asterisk (*) indicates significant correlations after BH-FDR correction (q < 0.05). Red: positive correlations; blue: negative correlations.

Figure 8 depicts the heatmap of the Spearman correlation analyses performed within the OVX intervention groups among SCFA concentrations, plasma parameters, muscle-related gene markers, and gut microbiota composition. The three major SCFAs, acetate, propionate, and butyrate, were negatively correlated with TG and AUC, positively correlated with relative grip strength (forelimb and four-limb), and muscle synthesis-related gene expressions (GLUT4, MyoG, IGF-1, IRS-1, PI3K, Akt, mTOR, S6K1, and eIF-4EBP1). In contrast, they were negatively correlated with the muscle atrophy- and inflammation-related gene expressions (FOXO1, MuRF-1, Atrogin-1, and IL-6). Acetate, propionate, and butyrate were positively correlated with phylum *Actinobacteriota* and genus *Lachnospiraceae_UCG_001*, whereas acetate and butyrate were positively correlated with genus *Muribaculum*. These correlations remained significant after BH-FDR correction (q < 0.05). These correlation findings should be interpreted as associative rather than mechanistic.

## 4. Discussion

The main metabolic findings of the present study were derived from comparisons within the age-matched OVX groups. In this context, HFD was associated with greater adiposity and impaired glucose-related outcomes, as reflected by increased visceral fat mass, glucose AUC, and HOMA-IR in the OH group compared with the OC group [32,33]. Partial substitution of the diet with FFRB attenuated obesity- and glucose intolerance-related indices. Trend analysis further showed significant dose-dependent reduction in body weight gain and insulin resistance markers, including fasting glucose, AUC, and HOMA-IR, across the intervention groups. Consistent with our findings, previous studies have reported that rice bran or its extract may suppress weight gain and improve plasma lipid and glucose profiles in HFD-fed mice [33,34,35]. The high insoluble fiber content of rice bran has also been associated with improved glycemic regulation [36]. Skeletal muscle is the primary site of insulin-stimulated glucose disposal [37]. Accordingly, increased muscle mass may enhance glucose uptake, thereby establishing a positive feedback loop that limits glucose diversion toward de novo lipogenesis and contributes to reduced fat mass. Thus, these findings suggest that FFRB, rich in indigestible dietary fiber, was associated with improvements in body weight, adiposity, lipid metabolism, and glucose homeostasis in HFD-fed OVX mice.

Skeletal muscle is highly responsive to insulin, facilitating glucose uptake from the bloodstream via GLUT4 transporters, and storing it as glycogen, so playing a crucial role in maintaining glucose homeostasis [37]. Insulin signaling in skeletal muscle is also crucial for regulating the balance between protein synthesis and degradation to preserve muscle mass. FFRB intervention attenuated the decline in muscle-related outcomes, particularly in the OHR20 group. Regarding the different responses observed between the forelimb and four-limb grip strength tests, these two assays involve different limb engagement and load-bearing characteristics. The forelimb test primarily reflects upper limb performance, whereas the four-limb test engages both forelimbs and hindlimbs and is more influenced by body weight support and postural stability. Since the muscle analyzed in this study (GAS) is located in the hindlimb, the four-limb grip strength test is more directly related to the functional status of the target muscle group. Studies have shown that forelimb, hindlimb, and combined all-limb grip strength measurements are not interchangeable and may differ in their sensitivity to functional changes [38,39]. Accordingly, the differing trends observed between forelimb and four-limb grip strength in our study may reflect the different limb involvement and load-bearing characteristics of these two assays. Our findings also revealed a dose-dependent upregulation of insulin sensitivity and protein synthesis genes (IRS-1, mTOR, and eIF-4EBP1), and downregulation of muscle atrophy markers (FOXO1, MuRF-1) and the inflammatory cytokine IL-6 with increasing FFRB dosage. Previous reports showed that HFD decreased IRS-1, mTOR, p4EBP1, and S6K expressions in rodents [40,41], reduced MyoG expression in aged obese female mice [42], and increased muscle atrophy markers (atrogin-1, MuRF-1) while enhancing proinflammatory responses [26,43,44]. Such changes promote protein degradation and contribute to muscle loss. Previously published data demonstrated that semi-defatted rice bran and whole grain cereals mitigate muscle atrophy in HFD-fed mice by enhancing muscle protein synthesis and reducing proteolytic activity [26,45]. In line with these studies, our findings showed that 20% FFRB was associated with higher myogenesis gene MyoG and markers related to the mTOR/eIF-4EBP1 pathway, while lower IL-6, FOXO1, and MuRF-1. Therefore, the current study suggests that FFRB may be associated with a muscle-related profile consistent with reduced inflammation, lower FOXO1-related proteolysis, and improved muscle proteostasis in HFD-fed OVX mice.

Aging and HFD are well-established modulators of the gut microbiome. The gut microbiota, particularly in the large intestine, is a key source of lipopolysaccharides (LPS) contributing to metabolic endotoxemia [46,47]. Fecal microbiota transplantation from HFD-fed mice to germ-free mice has been shown to elevate NF-κB activation and induce intestinal inflammation [48]. Inflammation driven by cytokines like TNF-α may disrupt intestinal barrier-related function by activating NF-κB and reducing ZO-1 expression [49]. SCFAs are critical for maintaining intestinal barrier integrity. Tight junction proteins such as claudin-1, ZO-1, and occludin regulate gut permeability and help protect against inflammation-induced barrier disruption [50,51]. Aging and HFD have been shown to reduce colonic ZO-1 and occludin expression [52,53]. Our observation showed that adult HFD-fed OVX mice showed a less favorable colonic tight junction-related gene expression profile. We identified a significant positive trend in colonic ZO-1 and occludin expression across increasing FFRB doses. This suggests that FFRB was associated with improved intestinal barrier-related gene expression. This finding aligns with previous studies showing associations between rice bran intake and improved barrier-related outcomes [26,54]. This improvement in barrier function coincided with higher fecal acetate, propionate, and butyrate concentrations at the study endpoint, particularly the dose-dependent increase in acetate concentrations. Published studies indicated that dietary fiber intake was positively correlated with SCFA levels, especially acetate, butyrate, and propionate [55,56].

The gut microbiota is closely linked to SCFAs production through fermentation of dietary fibers [15], with families such as *Lactobacillaceae*, *Ruminococcaceae*, and *Lachnospiraceae* being key contributors [57]. Our endpoint analysis showed that *Lachnospiraceae* were positively associated with fecal acetate, propionate, and butyrate concentrations, while *Ruminococcaceae* contributed less prominently. These findings may be consistent with the possibility that FFRB, as a fiber-rich ingredient, is associated with gut-related patterns linked to fecal SCFA concentrations. However, because no microbiota features remained statistically significant after BH-FDR correction in the one-way comparison, and because baseline microbiota and SCFA data were not available, these microbiota–SCFA patterns should be interpreted cautiously as descriptive and cross-sectional endpoint relationships rather than direct evidence of FFRB-induced temporal shifts.

These microbiota–SCFA interactions may have broader implications for both metabolic health and skeletal muscle. Butyrate and propionate exert anti-inflammatory effects and promote glucose regulation, while SCFAs in general are linked to muscle protein synthesis and strength [58]. In our study, fecal SCFAs were positively correlated with grip strength (forelimb and four-limb) and with the expression of muscle anabolic genes (GLUT4, MyoG, IGF-1, IRS-1, PI3K, Akt, mTOR, S6K1, and eIF-4EBP1), while negatively correlating with muscle atrophy/inflammatory markers (FOXO1, MuRF-1, Atrogin-1, and IL-6). In addition, *Lachnospiraceae_UCG-001* and *Muribaculum* were associated with selected fecal SCFA concentrations in the correlation analysis. These observations suggest an associative relationship among selected gut-related features, fecal SCFAs, and muscle-related outcomes. However, these correlations were restricted to the intervention groups to evaluate associations within a shared pathological background, and were based on cross-sectional endpoint data; they should be interpreted as exploratory rather than causal.

Our study used different intestinal compartments to perform microbiota profiling and SCFA quantification. Cecal luminal contents were used for microbiota analysis because the cecum contains abundant microbial biomass and is often considered a robust site for characterizing distal gut microbial community structure in mice [59]. In contrast, fecal samples were used for SCFA analysis because they reflect the final excreted pool of fermentation products and are commonly used for SCFA quantification [60]. Because these samples were obtained from anatomically distinct compartments, direct one-to-one correspondence between bacterial abundance and SCFA concentrations should be interpreted with caution. Together, these findings suggest that FFRB consumption was associated with favorable endpoint patterns in gut barrier-related markers, fecal SCFA profiles, metabolic function, and muscle-related outcomes in middle-aged OVX mice. Further longitudinal studies with baseline microbiota and SCFA measurements are needed to confirm causality.

The present study emphasizes the potential value of using whole FFRB, rather than defatted rice bran or isolated extracts, which may overlook the synergistic effects of its diverse bioactive components. Besides optimizing the functional potential of whole FFRB, it also contributes to sustainability by reducing food waste and carbon emissions. In the present HFD-fed OVX mouse model, FFRB was associated with favorable metabolic and muscle-related outcomes, together with concurrent changes in gut-related parameters. As a dietary fiber-rich food, FFRB may represent a promising non-pharmacological nutritional approach for supporting metabolic and muscle-related health under estrogen-deficient conditions. These results provide preclinical evidence supporting FFRB as a sustainable, low-cost nutritional strategy in the context of obesity, glucose intolerance, and muscle atrophy.

There are several limitations in the present study. First, we acknowledged the age difference between the YC and the adult OVX groups as a limitation. However, this design was intentionally selected to provide a life-stage reference contrast between young sham-operated mice and adult OVX mice, reflecting the combined differences in age and ovarian status. Therefore, differences between YC and OC should be interpreted descriptively and not as evidence of a specific effect of aging or estrogen deficiency alone. Crucially, all FFRB intervention comparisons were performed strictly within age-matched OVX mice to ensure internal validity. Second, glucose tolerance (IPGTT), fecal SCFA concentrations, and gut microbiota composition were assessed only at the end of the intervention period, without baseline measurements. Although the OVX mice were of the same age, had similar baseline body weight, received the same diet before the intervention period, and were maintained under the same experimental conditions, these design features could only reduce, but not eliminate, potential pre-existing variation and do not replace baseline assessment. Therefore, the findings of this study should be interpreted as endpoint-associated differences rather than definitive intervention-induced changes. Future studies should incorporate baseline and longitudinal measurements, where feasible, to better clarify temporal changes and strengthen causal inference regarding the relationships among FFRB consumption, glucose tolerance, microbiota-related outcomes, and SCFA concentrations. Third, we did not measure circulating SCFA levels; however, we assessed the fecal SCFAs, which are considered a more direct reflection of gut microbial fermentation and activity in the large intestine [61]. Fourth, we did not characterize the bioactive compounds of FFRB, such as γ-oryzanol or phenolic acids, which are recommended for inclusion in future studies to provide a more comprehensive understanding of its functional properties. Fifth, we assessed muscle strength and gene expression markers, but did not examine mitochondrial energy metabolism. Given its central role in muscle function and insulin sensitivity, future studies should investigate whether FFRB or its derived SCFAs influence mitochondrial bioenergetics to further clarify the underlying mechanisms [16,41].

## 5. Conclusions

FFRB (TNG81) intervention in HFD-fed bilateral OVX mice was associated with enhanced intestinal barrier-related gene expression, including upregulation of colonic tight junction markers. FFRB was also associated with higher fecal SCFA concentrations at the study endpoint and exploratory gut-related differences. These changes were accompanied by improvements in insulin sensitivity, greater skeletal muscle strength, lower inflammatory markers, and lower muscle proteolytic gene expression. These findings support the potential of FFRB (TNG81) as a dietary strategy to promote metabolic and muscle-related health in HFD-fed OVX mice.

## Figures and Tables

**Table 1:** Diet composition of experimental groups.

	YC	OC	OH	OHR5	OHR10	OHR20
Energy (kcal)	3808.00	3808.00	4808.00	4842.40	4874.30	4943.60
Protein (kcal%)	14.90	14.90	11.81	11.73	11.65	11.57
Fat (kcal%)	9.50	9.50	44.93	44.63	44.37	43.62
Carbohydrate (kcal%)	75.60	75.60	43.26	43.63	43.98	44.81
Ingredients (g/kg)						
Cornstarch	465.0	465.0	265.0	244.0	222.5	182.0
Maltodextrin	155.0	155.0	155.0	155.0	155.0	155.0
Sucrose	100.0	100.0	100.0	100.0	100.0	100.0
Casein	140.0	140.0	140.0	133.0	125.9	112.8
L-Cysteine	2.0	2.0	2.0	2.0	2.0	2.0
Soybean oil	40.0	40.0	120.0	113.0	106.0	91.0
Lard	0.0	0.0	120.0	120.0	120.0	120.0
Cellulose	50.0	50.0	50.0	38.7	27.5	5.0
Mineral Mix (AIN-93M-MIX)	35.0	35.0	35.0	31.7	28.3	21.6
Vitamin Mix (AIN-93M-MIX)	10.0	10.0	10.0	9.5	9.0	8.0
Choline Bitartrate	3.0	3.0	3.0	3.0	3.0	3.0
Tert-butylhydroquinone	0.008	0.008	0.008	0.008	0.008	0.008
Rice bran	0.0	0.0	0.0	50.0	100.0	200.0
Total	1000.008	1000.008	1000.008	1000.400	1000.200	1000.400

Cornstarch: MP Biomedicals, Cat. No. 90295625, Solon, CA, USA; maltodextrin: MP Biomedicals, Cat. No. 96004801, Solon, CA, USA; sucrose: Taiwan Sugar Corp., Tainan, Taiwan; casein: MP Biomedicals, Cat. No. 90129325, Solon, CA, USA; L-cysteine: MP Biomedicals, Cat. No. 10145480, Solon, CA, USA; soybean oil: Taiwan Sugar Corp., Tainan, Taiwan; lard: I-Mei Foods Co., Ltd., Tainan, Taiwan; cellulose: MP Biomedicals, Cat. No. 90045305, Solon, CA, USA; AIN-93M mineral mix: MP Biomedicals, Cat. No. 96040102, Solon, CA, USA; AIN-93M vitamin mix: MP Biomedicals, Cat. No. 96040201, Solon, CA, USA; choline bitartrate: UNI-ONWARD Corp., Cat. No. SI-C1629, New Taipei City, Taiwan; tert-butylhydroquinone: UNI-ONWARD Corp., Cat. No. AL-112941, New Taipei City, Taiwan.

**Table 2:** Nutrient contents of Tainung No. 81 full-fat rice bran.

Nutrient Contents	100 g
Energy (kcal)	418.7
Protein (g)	14.1
Fat (g)	14.3
Carbohydrate (g)	58.4
Soluble fiber (g)	1.6
Insoluble fiber (g)	20.9
Sugars (g)	5.2
Sodium (mg)	5.4
Moisture (g)	6.5
Ash (g)	6.7

**Table 3:** Amino acid contents in Tainung No. 81 full-fat rice bran.

Amino Acids	mg/kg	Amino Acids	mg/kg
Alanine	7870	Lysine	5935
Arginine	9678	Methionine	1662
Aspartic acid	13,452	Phenylalanine	5815
Cystine	1582	Proline	6560
Glutamic acid	18,758	Serine	5557
Glycine	6318	Threonine	4743
Histidine	4374	Tyrosine	2557
Isoleucine	5133	Valine	7389
Leucine	9016	Tryptophan	1540

## Data Availability

The original data presented in this study are openly available in Figshare at https://doi.org/10.6084/m9.figshare.32056224.

## Supplementary Materials

The following supporting information can be downloaded at: https://www.mdpi.com/article/10.3390/nu18111774/s1, Table S1: Primer sequences used for real-time quantitative polymerase chain reaction analysis; Table S2: GC program and operating conditions.

## Abbreviations

The following abbreviations are used in this manuscript:

Akt	Alpha serine/threonine-protein kinase
AUC	Area under the curve
BH	Benjamini–Hochberg
eIF-4EBP1	Eukaryotic translation initiation factor 4E binding protein 1
FDR	False discovery rate
FER	Food efficiency ratio
FFRB	Full-fat rice bran
FOXO1	Forkhead box protein O1
GAS	Gastrocnemius
GLU	Glucose
GLUT4	Glucose transporter type 4
HFD	High-fat diet
HOMA-IR	Homeostasis model assessment of insulin resistance
IGF-1	Insulin-like growth factor 1
IL-6	Interleukin 6
IPGTT	Intraperitoneal glucose tolerance test
IRS-1	Insulin receptor substrate 1
LDA	Linear discriminant analysis
LEfSe	Linear discriminant analysis effect size
mTOR	Mammalian target of rapamycin
MuRF-1	Muscle RING-finger protein-1
MyoG	Myogenin
OVX	Ovariectomized
PI3K	Phosphatidylinositol3-kinase
RT-qPCR	Real-time quantitative polymerase chain reaction
S6K1	Ribosomal protein S6 kinase beta-1
SCFAs	Short-chain fatty acids
SD	Standard deviation
TC	Total cholesterol
TG	Triglycerides
TNG81	Tainung No. 81
ZO-1	Zonula occludens-1
