# Supplementary material for: Full-Fat Rice Bran Ameliorates Insulin Resistance and Modulates Muscle-Related Parameters in High-Fat Diet-Fed Ovariectomized Mice with Potential Involvement of the Gut–Muscle Axis
Source: Nutrients. 2026 May 30;18(11):1774. doi: 10.3390/nu18111774 (PMC13259252; doi:10.3390/nu18111774)
Supplement: Supplementary file 1 [file nutrients-18-01774-s001.zip › nutrients-4302442-supplementary.pdf]

Supplementary Table S1. Primer sequences used for real-time quantitative polymerase chain reaction analysis.

Gene Name	Primer sequence (5'-3') Forward	Reverse
GAPDH	AACGACCCCTTCATTGAC	TCCACGACATACTCAGCAC
GLUT4	ATTGCAGCGCCTGAGTCTTT	GAGGGGGTTCCCCATCCTTA
MyoG	AGTGAATGCAACTCCCACAG	CTGGGAAGGCAACAGACATA
IGF-1	CAATACAGCCAACGGGAAACAG	AACAAAGCTGGATGCCTGTCA
IRS-1	CCGGATACCGATGGCTTCTC	CCGCCACTTCTTCTCGTTCT
PI3K	GGGAGCAGCCTGGATGATTT	AGCGATTGGTTCCCCACAAT
Akt	GCTTGCGGTCTGATGTTTTCT	GCCTTTTCCAGCCACAAACA
mTOR	CGTCACAATGCAGCCAACAA	TGCCTTTCACGTTCCTCTCC
S6K1	ACACCCTCCATCCTGGAGTAA	TTGTTCACGATAAGTCTCCACCT
eIF-4EBP1	TCACTAGCCCTACCAGCGAT	TTGTGACTCTTCACCGCCTG
FOXO1	CGGAAAATCACCCCGGAGAA	TACACCAGGGAATGCACGTC
Atrogin-1	AACCGGGAGGCCAGCTAAAGAACA	TGGGCCTACAGAACAGACAGTGC
MuRF-1	CCTTGAGGGCCATTGACTTG	TCCCCTCAGAACTCAAGAGGAA
IL-6	GTCCTTCCTACCCCAATTTCCA	TGGTCTTGGTCCTTAGCCAC
ZO-1	GGCACATCAGCACGATTTCT	CCACAAAAGAAATCCTTTCACACCT
Occludin	ACTGGGTCAGGGAATATCCA	TCAGCAGCAGCCATGTACTC

Supplementary Table S2. GC program and operating conditions.

GC program	Conditions
Injection location	Front
Injection volume	1 μL
Solvent A washes	Ethyl acetate, 2 μL, 2 times pre-injection, 2 times post-injection
Sample washes	2 μL, 2 times pre-injection
Sample pumps	2
Plunger speed	Slow
Inlet temperature	200 °C
Inlet pressure	16.288 psi
Inlet mode	Pulsed split
Split flow	60 mL/min
Column flow	Constant flow
Post run	1.2 mL/min (same as set point flow)
Capillary column	Nukol™ 30 m × 0.25 mm × 0.25 μm
Carrier gas type	Helium
Oven temperature	Initial: 90 °C<br>Ramp 1: rate 15 °C/min; value 150 °C; hold time 1 min<br>Ramp 2: rate 3 °C/min; value 170 °C; hold time 2 min<br>Ramp 3: rate 50 °C/min; value 200 °C; hold time 2 min<br>Post run: 90 °C
AUX heaters	200 °C
